# The Fragile Path: Resilience and Frailty Dynamics in Hip Fracture Recovery

**DOI:** 10.1093/geroni/igaf122.1057

**Published:** 2025-12-31

**Authors:** Chenkai Wu, Jianhong Xu, Yanxin Wang, Jonathan Ka-Long Mak, Emiel O Hoogendijk, Graciela Muniz-Terrera

**Affiliations:** Duke Kunshan University, Kunshan, Jiangsu, China (People’s Republic); Duke Kunshan University, Kunshan, Jiangsu, China (People’s Republic); Duke Kunshan University, Kunshan, Jiangsu, China (People’s Republic); The University of Hong Kong, Hong Kong, China (People’s Republic); VU University Medical Center, Amsterdam, Noord-Holland, Netherlands; University of Edinburgh, Edinburgh, Scotland, United Kingdom

## Abstract

Hip fractures represent a significant stressor, revealing underlying resilience deficits, particularly in frail individuals. This study examines frailty trajectories surrounding hip fracture and their joint impact on mortality. Using data from 4,963 UK Biobank participants with incident hip fracture, we assessed frailty monthly via the Hospital Frailty Risk Score (derived from ICD-10 codes) for 12 months before and after hip fracture. Latent class trajectory models identified distinct frailty trajectories 12 months before and after hip fracture, separately. We used the multinomial logistic regression to examine transitions between pre- and post-fracture frailty trajectories. Survival analysis was used to determine the association between the trajectories and death. We identified three frailty trajectories in the 12 months before hip fracture: persistently robust (44.2%), moderately frail (37.1%), and progressively frail (18.7%). Frailty remained stable except in the progressively frail group, where frailty scores increased by 28.6% in the year before fracture. After hip fracture, three distinct trajectories emerged: moderate frailty with stability (63.0%), high frailty with stability (30.8%), and rapidly progressive frailty (6.2%). Higher pre-fracture frailty was strongly linked to greater post-fracture frailty severity. Pre- and post-fracture frailty trajectories were independently and jointly associated with death; higher pre-fracture frailty was linked to higher mortality within the same post-fracture category. These findings demonstrate distinct frailty trajectories surrounding hip fracture, highlighting the predictive value of pre-fracture frailty for post-fracture outcomes and mortality. Identifying high-risk individuals through these trajectories can inform targeted interventions to improve recovery and survival.

